# Machinability of Different Wood-Plastic Composites during Peripheral Milling

**DOI:** 10.3390/ma15041303

**Published:** 2022-02-10

**Authors:** Zhaolong Zhu, Dietrich Buck, Jinxin Wang, Zhanwen Wu, Wei Xu, Xiaolei Guo

**Affiliations:** 1 Co-Innovation Center of Efficient Processing and Utilization of Forest Resources, Nanjing Forestry University, Nanjing 210037, China; njfuzzlong@outlook.com (Z.Z.); xuwei@njfu.edu.cn (W.X.); 2 College of Furnishings and Industrial Design, Nanjing Forestry University, Nanjing 210037, China; 3 Wood Science and Engineering, Luleå University of Technology, 931 87 Skellefteå, Sweden; dietrich.buck@ltu.se; 4 College of Materials Science and Technology, Nanjing Forestry University, Nanjing 210037, China; jackiewang@njfu.edu.cn (J.W.); wuzhanwen@njfu.edu.cn (Z.W.)

**Keywords:** Taguchi method, optimization, WPC, milling, machinability

## Abstract

The aim of this study was to improve the machinability of wood-plastic composites by exploring the effects of different wood-plastic composites on machinability. In particular, the effects of milling with cemented carbide cutters were assessed by investigating cutting forces, cutting temperature, surface quality, chip formation, and tool wear. The cutting parameters determined to yield an optimal surface quality were rake angle 2°, cutting speed 9.0 m/s, feed per tooth 0.3 mm, and cutting depth 1.5 mm. In these optimized milling conditions, the wood-plastic composite with polypropylene exhibited the highest cutting forces, cutting temperature, and tool wear, followed by polyethylene and polyvinyl chloride wood-plastic composites. Two wear patterns were determined during wood-plastic composite machining, namely chipping and flaking. Due to the different material composition, semi-discontinuous ribbon chips and continuous ribbon chips were generated from the machining process of wood-plastic composites with polypropylene and polyethylene, respectively. The wood-plastic composite with polyvinyl chloride, on the other hand, formed needle-like chips. These results contribute to a theoretical and practical basis for improved wood-plastic composite machining in industrial settings.

## 1. Introduction

Wood-plastic composites (WPC) made by mixing wood fiber or wood dust with plastic followed by extrusion and hot-pressing are an environmentally friendly alternative to traditional building materials [1,2]. With excellent inherent fire resistance and processability making them compatible with a variety of industrial molding and infrastructure, WPCs find applications in interior and exterior markets pertaining to furnishing, decorating, and packing, to name a few [3,4]. WPCs can be of different types depending on their plastic component, such as polypropylene (WPPC), polyethylene (WPEC), or polyvinyl chloride (WPVCC) [5,6,7]. Unlike plastics, for which production is primarily through extrusion, WPC products are manufactured using drilling, planing, turning, and milling [8,9]. This leads to issues pertaining to cutting forces and temperature, surface quality, chip formation, and tool wear, much like the case of traditional timber. Understanding the effects machining processes have on WPC products is critical, not only to create better quality products, but also to enhance tool longevity. Thus, it is not surprising that many researchers focus on understanding these aspects of WPC machining.

In the recent past, effects of cutting forces, heat, machined surface quality, chip formation, and tool wear on the machinability of WPC were assessed [10]. It is important to note that tool geometries and certain cutting parameters also influence these effects. For instance, Guo et al. [11] explored the changes in cutting forces and surface roughness during orthogonal machining of WPC and found that they were positively related to cutting depth. Their results also confirmed that when the cutting depth was low, the process resulted in curly and continuous chips. In a subsequent study, it was determined that chips resulting from orthogonal cutting of WPC could exhibit four distinct morphologies: short continuous, long continuous, granule, and flake. Shi et al. [12] investigated variations in chip size during WPC milling and found that while thickness was positively correlated with feed speed, the opposite was true for cutting speed. Through a systematic study which considered multiple process factors, Pei et al. [13] confirmed that cutting depth contributed most to cutting temperature, followed by spindle speed and cutting width. Interestingly, they also found that most of the heat generated during cutting was taken away by the chips generated. In yet another study investigating surface quality of WPC subjected to turning, Hutyriva et al. showed that cutting quality increased when high spindle speeds and slow feed rates were used [14]. In a related study, a series of carefully designed drilling experiments revealed that surface defects on WPC are strongly linked to tool geometries and material properties [15].

When it comes to industrial machining, cutting WPC is challenging owing to the diverse types of plastics they contain. Irrespective of the type of WPC, plastics inherently have low thermal stability and are easily affected by the heat generated during cutting processes. This thermal-mechanical coupling leads to knockdown effects on the overall precision of the machining process and final surface quality of WPC products [16]. To make things worse, WPCs are two to four times denser than traditional wood-based materials, which increases the wear rate of the cutting tool considerably [17]. Considering WPC is a relatively new material, extensive research exploring key machining parameters to optimize its machinability and improve manufacturing yield is the need of the hour.

Thus, this study’s objective was to identify possible relations between cutting forces, cutting temperature, surface roughness, chip formation, and tool wear pertaining to WPC machining with cemented carbide cutters. Specifically, optimized cutting parameters for three types of WPC, namely, WPPC, WPEC, and WPVCC, were determined by assessing surface finish quality and tool wear, amongst others. These findings can provide scientific guidance to improve WPC machining practices in the industry.

## 2. Materials and Methods

### 2.1. Workpiece and Cutting Tool

Table 1 lists the three types of WPCs, namely, WPPC, WPEC, and WPVCC (Guofeng Wood-Plastic Composite Company Co., Ltd., Anhui, China) used in this study. All three types of WPC were obtained from 4:5 mixtures of the plastic and wood fiber and were processed by extrusion, molding, and injection molding. Details of the material composition and properties of all WPCs prepared for this work are provided in Table 1. Cutters used to machine WPC were supplied by Leitz Tooling System Co. Ltd. (Nanjing, China). The inserts fixed on the cutter’s body comprised tungsten carbide, cobalt, and other compounds. Table 2 presents the tool geometries and tool material properties.

### 2.2. Experimental Set-Up

**Figure 1:**
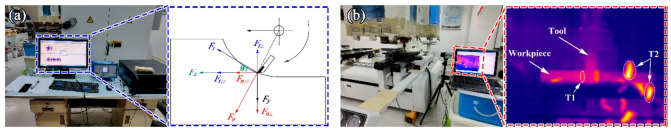
Measurements of (**a**) force by piezoelectric dynamometer, (**b**) cutting temperature by infrared imaging system.

Up milling was performed on a computerized numerical control machine (MGK01, Nanxing Machinery Co., Ltd., Dongguan, China) in dry conditions. As shown in Figure 1a, dynamic cutting force signals were monitored using a three-dimensional piezoelectric dynamometer (Kistler 9257B, Switzerland) with a charge amplifier (5070A, Switzerland) connected to a computer. In this work, straight tooth cutting tools were used, and only the tangential forces (*F_t_*) and normal forces (*F_n_*) were studied. These forces were calculated based on Equations (1) and (2):

(1) $$\theta =\mathrm{arc}sin\sqrt{\frac{a_{p}}{D}}$$

(2) $$\left(\begin{array}{l} F_{t}\\ F_{n} \end{array}\right)=\left(\begin{array}{l} \cos\theta \\ \sin\theta \end{array}\right.\;\left.\begin{array}{l} -\sin\theta \\ \cos\theta \end{array}\right)\left(\begin{array}{l} F_{x}\\ F_{y} \end{array}\right)$$

where *θ* denotes the angle between the feeding and rotation directions in °; *a_p_* is the cutting depth in mm; *D* represents the tool diameter of 18 cm; *F_t_* and *F_n_* are defined as the force components perpendicular and parallel to the radius direction in N, respectively (see Figure 1a inset); and *F_x_* and *F_y_* are the measured forces parallel and perpendicular to the feeding direction in N, respectively (see Figure 1a inset).

An infrared imaging camera with an emissivity of 0.9 (Figure 1b, A20-M, Thermo Fisher Co. Ltd., Waltham, MA, USA) was used to measure and monitor the dynamic cutting temperature during milling. The dynamic temperature of the cutting-edge of the tool and the chips are represented by the characters *T_1_* and *T_2_*, respectively (Figure 1b inset), while the room temperature was 24 °C. Furthermore, surface roughness *Ra* was used to evaluate the smooth machined surface, and was measured using a surface profilometer with sensitivity of 0.1–20 nm (S-NEX001SD-12, Olympus, Co. Ltd., Tokyo, Japan). Finally, morphologies of chips and the extent of tool wear were observed using a scanning electron microscope (Quanta 200, FEI Group Co. Ltd., Hillsboro, OR, USA). Finally, radius of the tool edge was measured with an optical microscope (SZX16, Olympus Co. Ltd., Tokyo, Japan).

### 2.3. Experimental Design

In this work, two groups of milling experiments, designated as Experiments I and II, were designed. Experiment I was used to determine a set of optimal cutting parameters which result in the lowest surface roughness values for cut specimens. Experiment II was designed to investigate the effect of different kinds of plastic contained in WPC on the machinability of WPC, using the optimal cutting parameters obtained from Experiment I.

In Experiment I, the selection of experimental cutting factors shown in Table 3 was based on industrial WPC machining best practices and the Taguchi method [18]. An L_27_ (4^3^) orthogonal array was adopted, with three different levels of rake angle *α* (°), cutting speed *v* (m/s), feed per tooth *U_z_* (mm/Z), and cutting depth *a_p_* (mm). Calculated signal-to-noise ratio (*SNR*) values were used to determine a group of optimal cutting parameters derived from the experimental cutting factors. These optimal cutting parameters are expected to yield the lowest surface roughness *Ra*, i.e., the lowest value of *SNR* will correspond to the highest cutting quality. The equation to obtain *SNR* values is Equation (3) [19]:

(3) $$SNR=-10\cdot \mathrm{lg}\sum_{i=1}^{n}\frac{y_{i}{}^{2}}{n}$$

where *n* is the testing number and *y_i_* is the *Ra* value of each testing.

In Experiment II, the machinability of WPPC, WPEC, and WPVCC using the optimal cutting parameters was investigated. The machinability parameters monitored included cutting forces, cutting temperature, chip formation, and tool wear.

## 3. Results and Discussion

### 3.1. Optimal Cutting Parameters for Wood-Plastic Composite Machining

Results of *SNR* values calculated using Equation (3) are provided in Table 4. A higher delta value means a more pronounced contribution of the experimental cutting factor to the surface roughness. Bearing this in mind, feed per tooth gave a delta value of 0.982 (Rank 1), followed by cutting depth (delta = 0.539, Rank 2), cutting speed (delta = 0.292, Rank 3), and rake angle (delta = 0.192, Rank 4). Thus, feed per tooth exhibits the strongest influence on surface roughness, followed by cutting depth, cutting speed, and rake angle, in that order.

**Figure 2:**
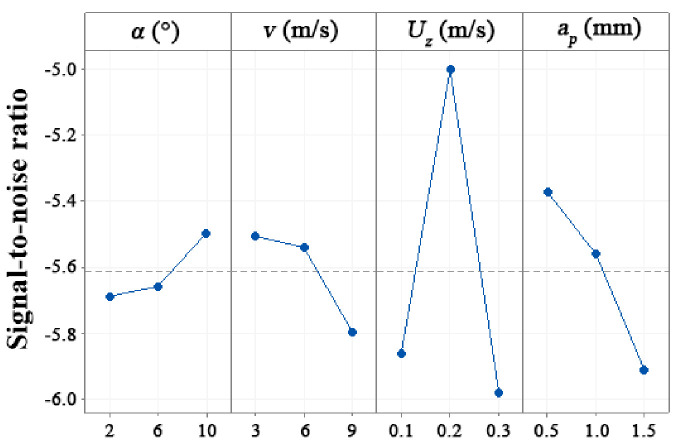
Experiment I: main effects of the experimental cutting factors on SNR values for surface roughness.

The effects of all these experimental cutting factors on *SNR* values pertaining to surface roughness are shown in Figure 2. Based on the determined *SNR* values for *Ra* and their respective ranks, the optimal combination of cutting parameters was determined to correspond with the lowest value of *SNR* for each experimental cutting factor. The optimal cutting parameters determined in this study were rake angle 2°, cutting speed 9.0 m/s, feed per tooth 0.3 mm, and cutting depth 1.5 mm. This is in contrast to traditional wood materials [20,21,22,23]. Optimal cutting parameters for WPC yielding the lowest surface roughness were determined to include a higher rake angle and cutting speed, at a lower feed per tooth and cutting depth. This phenomenon is thought to be caused by the plastic contained in WPCs. With a decrease in rake angle and an increase in cutting speed, feed per tooth, and cutting depth, higher friction may be generated between the WPCs and cutters, which leads to a higher cutting temperature. The heat makes the WPC less rigid, resulting in the edges of the WPC, which are more susceptible to penetration, being shorn off.

### 3.2. Effects of Wood-Plastic Composite Types on Cutting Forces

**Figure 3:**
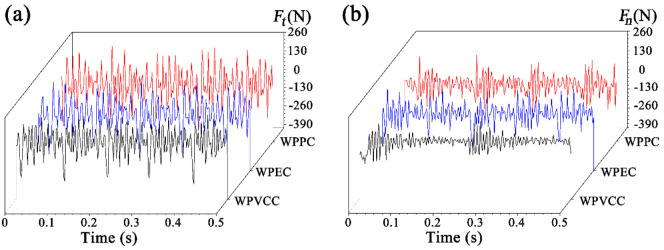
Experiment II: changes in dynamic cutting forces of (**a**) *F_t_* and (**b**) *F_n_* with different WPC types.

Figure 3 shows the tangential and normal dynamic cutting forces (*F_t_* and *F_n_*) for WPPC, WPEC, and WPVCC at the optimal combination of cutting parameters (*α* = 2°, *v* = 9.0 m/s, *U_z_* = 0.3 mm, and *a_p_* = 1.5 mm). The data indicate that the tangential force was consistently higher than the normal force, irrespective of the type of WPC. Furthermore, WPPC had the highest cutting forces (*F_t_* and *F_n_*) under the same cutting parameters, followed by WPEC and WPVCC. As seen in Table 1, WPPC also exhibits the highest flexural strength, modulus of elasticity, and density, followed by WPEC and WPVCC. Thus, it seems logical that during processing of the sturdier material, the cutting edge encountered greater resistance from the workpiece, leading to higher tangential and normal forces. Accordingly, it was found that the highest cutting forces were generated during machining of WPPC, followed by WPEC and WPVCC.

### 3.3. Effects of Wood-Plastic Composite Types on Cutting Temperature

**Figure 4:**
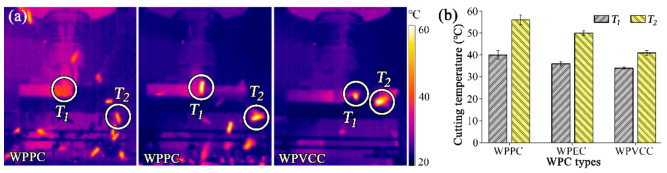
Experiment II: changes in (**a**) dynamic cutting temperature and (**b**) maximum cutting temperature with different WPC types.

The changes in maximum cutting edge temperature (*T_1_)* and chip temperature (*T_2_*) of different types of WPC milled at the optimal combination of cutting parameters (*α* = 2°, *v* = 9.0 m/s, *U_z_* = 0.3 mm, and *a_p_* = 1.5 mm) are presented in Figure 4. Overall, regardless of the type of WPC, the chip temperature was higher than the cutting edge temperature. During material machining, heat is mainly produced from friction and phase change of the plastic. As the cutting tool removes the unwanted material, chips take away most of the heat, while a small fraction of the heat remains on the tool surface and is dissipated into ambient air. This finding agrees with the work by Pei et al. [13], which investigated cutting heat of WPC milling. Furthermore, the temperatures of the cutting edge and the chips had trends similar to the cutting forces; WPPC exhibited the highest cutting temperature, followed by WPEC and WPVCC. This behavior is also attributed to the fact that WPPC was the sturdiest material of the lot, which meant higher resistance acted on the cutting tool, leading to higher cutting temperatures when compared to WPEC and WPVCC.

### 3.4. Chip Morphology

**Figure 5:**
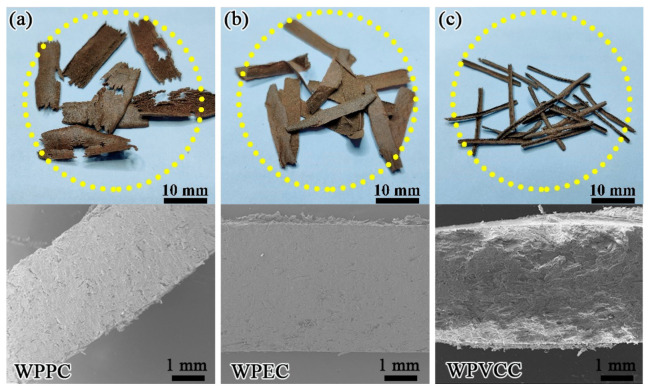
Experiment II: chip morphologies of (**a**) WPPC, (**b**) WPEC, and (**c**) WPVCC.

Cutting is a material removal process and as such, when the total force from the cutter exceeds the ultimate strength of the material of interest, material is removed by the cutter as chips of different shapes and sizes [9,10]. During WPC’s machining, only three types of chips were released at the optimal combination of cutting parameters (*α* = 2°, *v* = 9.0 m/s, *U_z_* = 0.3 mm, and *a_p_* = 1.5 mm), namely semi-discontinuous ribbon chips, continuous ribbon chips, and needle chips (Figure 5). For the most part, these chips are similar in shape to those generated during orthogonal cutting of WPCs [11]. Upon closer inspection, the chips of WPPC were in the shape of flakes, some of which were broken. All WPPC chips resembled semi-discontinuous ribbon chips. The chips from WPEC were shaped in a continuous flake-like form, and were defined as continuous ribbon chips. Interestingly, unlike the ribbon chips from WPPC and WPEC, the chip shapes of WPVCC were needle-like (Figure 5).

Based on research into plastic-based material properties, PVC is known to have a lower toughness than PP and PE. Thus, when WPVCC was milled, the cutting layer was quickly broken, leading to the generation of needle chips because of the low elastoplasticity of WPVCC. PP being much harder than PE led to the formation of ribbon chips with a semi-discontinuous shape.

### 3.5. Tool Wear

**Figure 6:**
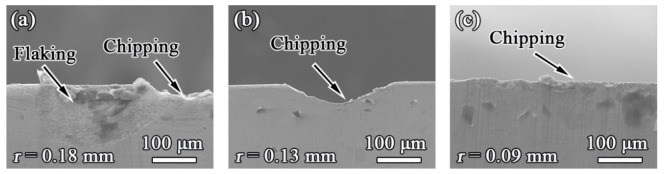
Experiment II: flank wear of cemented carbide cutters during machining of (**a**) WPPC, (**b**) WPEC, and (**c**) WPVCC.

Flank surface wear of the cemented carbide cutters used in this study was evaluated using scanning electron micrographs after a cutting distance of 500 m was completed (Figure 6). As can be clearly seen, the main wear patterns of cemented carbide cutters during machining of WPCs were chipping and flaking. Flaking is a severe wear pattern, wherein the original sharpness and appearance of the cutting edge change considerably, directly affecting the tool’s cutting stability and quality [24]. Furthermore, based on the degree of tool wear, it was observed that the radius of tool edge (*r* = 0.18 mm) when machining WPVCC was higher than those for WPEC (*r* = 0.13 mm) and WPPC (*r* = 0.09 mm). In other words, the wear of cutting tools when machining WPPC was more severe when compared with WPEC and WPVCC (Figure 6). It is known that tool wear is mainly affected by thermal-mechanical coupling [25]. As described in Section 2 in the present work, peripheral milling was adopted, which is an intermittent cutting process. Thus, the cutting edge periodically cuts into and withdraws from the workpiece. Repeated cycles of instantaneous impact force and periodic stress led to tool wear with different characteristics, namely flaking and chipping. As detailed above, the cutting forces and cutting temperatures when machining WPPC were both higher than those for WPEC and WPVCC. Consequentially, the most severe tool surface wear resulted from machining WPPC, followed by WPEC and WPVCC.

## 4. Conclusions

This work aimed to explore the cutting performance of wood-plastic composites based on cutting forces, cutting temperature, surface quality, chip formation, and tool wear. The main conclusions from this study based on a series of peripheral milling experiments using cemented carbide cutters are as follows:

(1) Optimal cutting parameters for the three WPCs machining yielding the lowest surface roughness are 2° rake angle, 9.0 m/s cutting speed, 0.3 mm feed per tooth, and 1.5 mm cutting depth.
(2) WPPC exhibited the highest cutting forces and cutting temperatures under the same cutting conditions, followed by WPEC and WPVCC.
(3) Three types of chips were formed during machining of the three types of WPC, namely continuous ribbon chips (WPEC), semi-discontinuous ribbon chips (WPPC), and needle chips (WPVCC).
(4) Tool wear when machining WPPC was more severe when compared to WPEC and WPVCC, with the dominant wear pattern for WPPC being chipping and flaking.

It is important to note that the focus of this work was on cutting forces, temperature, and chip and tool wear. Surface damage and morphology are also crucial parameters to evaluate the machinability of WPCs. In the future, special attention should be given to the machined surface to further increase value yield during the WPC manufacturing process.

## Figures and Tables

**Table 1:** Material composition and properties of wood plastic composites obtained by four samples of each type.

WPC Type	Material Composition		Material Properties			
	Plastic	WOOD FIBER	Moisture Content (%)	Flexural Strength (MPa)	Modulus of Elasticity (GPa)	Density (g/cm^3^)
WPPC	PP	Poplar	2.6	26.35	2.42	1.47
WPEC	PE		2.5	22.44	2.19	1.28
WPVCC	PVC		2.9	20.08	2.02	0.93

**Table 2:** Tool geometries and properties.

Tool Geometry			Material Properties		
Rake Angle	Clearance Angle	Cutter Diameter	Bending Strength	Thermal Conductivity	Hardness
2°	55°	18 cm	1.5 GPa	76.2 W·m^−1^·K^−1^	88.3 HRA
6°					
10°					

**Table 3:** Experimental cutting factors and levels.

Level	Experimental Cutting Factors			
	*α* (°)	*v* (mm/s)	*U*_z_ (mm/Z)	*a*_p_ (mm)
1	2	3	0.1	0.5
2	6	6	0.2	1.0
3	10	9	0.3	1.5

**Table 4:** Experiment I: SNR values of each experimental cutting factor during WPC milling at the different experimental cutting factor levels.

Level	*α* (°)	*v* (m/s)	*U*_z_ (mm/Z)	*a*_p_ (mm)
1	−5.689	−5.506	−5.861	−5.372
2	−5.658	−5.540	−5.000	−5.560
3	−5.496	−5.798	−5.982	−5.911
Delta	0.192	0.292	0.982	0.539
Rank	4	3	1	2

## Data Availability

Not applicable.
